# The shunt from hell: management based on the point of obstruction

**DOI:** 10.1186/2045-8118-12-S1-P45

**Published:** 2015-09-18

**Authors:** Harold L Rekate

**Affiliations:** 1 Hofstra Northshore LIJ School of Medicine, United States of America

## Introduction

After two years of study and conversation a group of 20 hydrocephalus investigators developed a consensus on a new classification of hydrocephalus based on point of obstruction. This new classification has proved useful in the diagnosis and treatment of patients with hydrocephalus of all types and is especially helpful in assessing the various treatment options for the individual patient.

## Methods

This is a retrospective review of a patient with a complex form of hydrocephalus related to a Dandy Walker Malformation who had had over 60 shunt revisions in the two years prior to first being seen at our insitution. She was evaluated as to potential points of obstruction and the best treatment for each point. The plan was to make certain that all compartments containing CSF were to be made to communicate with each other and that a maximum of one valve would be used if further treatment is needed.

## Results

Using injections of Iohexal tracer for CT it was shown that there were very small lateral and third ventricles and obstruction to flow from through the aqueduct and from the cortical subarachnoid space into the encysted fourth ventricle. Treatment progressed in stages with fenestration of the cyst and replacing the shunt with a reservoir and “Butterfly needle.” The lateral and third ventricle dilated and it was shown that now there was a fourth ventricle containing a floppy collapsed cyst within it. Following endoscopic third ventriculostomy she is now without a shunt and is back to school for the first time in 2 years. Follow up is 9 months.

## Discussion

The vast majority of cases of hydrocephalus are due to distinct sites of obstruction to flow of CSF. Normal CSF dynamics demand that all CSF compartements communicate freely and that the intracranial pressures are normal. At this point if the ventricles are able to increase in size the patient is likely (>80%) to be able to be managed without a shunt with or without an endoscopic third ventriculostomy.
